# Stage-specific and regional trends in prostate cancer incidence in Kazakhstan, 2005–2024

**DOI:** 10.3389/fonc.2025.1719720

**Published:** 2025-11-28

**Authors:** Gulnur Igissinova, Nurbek Igissin, Indira Kudaibergenova, Nariman Yermek, Zhansaya Telmanova, Dulat Turebayev, Akzhunis Jexenova, Rustem Moldagali, Gafur Khairli, Almas Kazhitaev, Sergey Dyakov, Daulet Baibosynov, Ivan Shishkin, Kalys Nogoibaeva, Altynai Baytelieva, Niiazbek Mamatov, Aram Halimi, Zarina Bilyalova

**Affiliations:** 1 Central Asian Institute for Medical Research, Astana, Kazakhstan; 2 Asfendiyarov Kazakh National Medical University, Almaty, Kazakhstan; 3 Research Institute of Life and Health Sciences, Higher School of Medicine, Kokshetau University named after Sh. Ualikhanov, Kokshetau, Kazakhstan; 4 Asian Pacific Organization for Cancer Prevention, Bishkek, Kyrgyzstan; 5 Akhunbaev Kyrgyz State Medical Academy, Bishkek, Kyrgyzstan; 6 Academy of Public Administration under the President of the Republic of Kazakhstan, Astana, Kazakhstan; 7 Astana Medical University, Astana, Kazakhstan; 8 Kokshetau Higher Medical College, Kokshetau, Kazakhstan; 9 National Scientific Center of Traumatology and Orthopedics named after Academician Batpenov N.D. of the Ministry of Health of the Republic of Kazakhstan, Astana, Kazakhstan; 10 Research Center for Social Determinants of Health, Research Institute for Metabolic and Obesity Disorders, Research Institute for Endocrine Sciences, Shahid Beheshti University of Medical Sciences, Tehran, Iran

**Keywords:** prostate cancer, Kazakhstan, epidemiology, incidence trends, PSA screening, joinpoint regression, stage distribution, morphological verification

## Abstract

**Background:**

Prostate cancer is a leading malignancy among men globally and continues to be a growing concern in Kazakhstan, where evidence regarding its long-term epidemiological trends is limited.

**Objectives:**

This study seeks to assess national and regional trends in prostate cancer incidence, stage distribution, and morphological verification in Kazakhstan from 2005 to 2024.

**Methods:**

A nationwide observational study utilizing registry data from the Unified Nationwide Electronic Health System was performed. Incident cases (ICD-10 code C61) were examined according to age, geographical region, and stage at diagnosis. The incidence rates were adjusted to the WHO World Standard Population (2000–2025) by age. Joinpoint regression was used to look at temporal trends, and it showed the annual percent change (APC) and the average annual percent change (AAPC) with 95% confidence intervals.

**Results:**

From 2005–2024, 21,756 prostate cancer cases were recorded, with a mean age at diagnosis of 69.8 years. The age-standardized incidence rate (ASR) increased from 11.9 to 20.7 per 100,000 men (APC = +2.6%, p = 0.002). Four distinct periods were identified: an early decline (2005–2008), a sharp rise (2008–2016), a downturn (2016–2020), and a renewed increase (2020–2024). Age-specific incidence was negligible below 50 years, peaking at 75–79 years (228.6 per 100,000). Regional analyses revealed heterogeneous trends: monotonic increases in Atyrau, Aktobe, and Almaty (region), contrasted by boom–dip–rebound profiles in Karaganda, Pavlodar, and Almaty City. The proportion of early-stage (I–II) cases rose from 32.8% to 56.9%, while stage III declined from 49.7% to 22.9%; stage IV increased slightly (17.3% → 20.2%). Morphological verification improved nationally (mean ≈ 92%) and plateaued after 2015.

**Conclusions:**

Kazakhstan shows an increase in prostate cancer cases, with more cases being diagnosed at an earlier stage but still a lot of cases being diagnosed at a later stage. This is probably due to the PSA screening program from 2013 to 2017, changes in policy since then, and problems with diagnosis during the pandemic. To get better results, we need to improve early detection, timely biopsy pathways, and connections to mortality and survival data.

## 1 Introduction

Prostate cancer remains a major global oncology challenge: according to GLOBOCAN 2022, it is the most commonly diagnosed cancer in men in most countries and ranks among the leading causes of cancer death (1). Accounting for population ageing and continuation of current incidence trends, The Lancet Commission projects a doubling of the global prostate cancer burden—from ~1.4 million new cases in 2020 to ~2.9 million by 2040 (2). Despite its frequency and impact on mortality, few lifestyle or environmental determinants have been established; advancing age, family history, and specific genetic alterations remain the only consistently validated risk factors, while proposed roles for smoking, excess adiposity, and selected dietary factors are suggestive but inconclusive (3).

Within Kazakhstan, national and regional analyses by local investigators documented rising incidence during 2007–2016—a period that overlaps temporally with the introduction of prostate-specific antigen (PSA)–based screening—while the largest cities (Almaty and Astana) consistently exhibited higher burdens than other regions (4–6). The 2013–2017 pilot population screening program demonstrated operational feasibility (hundreds of thousands of PSA tests) but also exposed critical limitations—from pre-analytical issues to the modest diagnostic specificity of PSA and the need for more judicious use of reflex indices/biopsy (7, 8). Local economic–epidemiological assessments (e.g., Pavlodar region) further underscored the sensitivity of outcomes to system capacity and patient routing (9). Against a background of limited public awareness, these factors can generate a "prevalence surge" at screening initiation and attenuating oscillations following program scale-down.

This highlights the pressing need for further study into the regional and temporal patterns of prostate cancer incidence and stage-specific trends. By addressing these gaps and study the effect of covid-19 pandemic on prostate cancer incidence in Kazakhstan, this study aims to provide critical data that can inform more targeted, evidence-based interventions, improving early detection and ultimately reducing the burden of advanced prostate cancer in Kazakhstan.

We undertook a comprehensive assessment of prostate cancer epidemiology in Kazakhstan over 2005–2024 with three emphases: a) national and regional incidence levels (age-standardized rates) and their age-specific profiles; b) stage distribution and temporal trends; and c) morphological verification as a key marker of data validity.

## 2 Materials and methods

### 2.1 Study design and setting

We conducted a nationwide, population-based, observational time-trend study of prostate cancer in Kazakhstan for calendar years 2005-2024. The unit of analysis was the male resident population at national and regional levels. Regions were defined according to the administrative structure used by the registry for longitudinal consistency (including the two cities of republican significance).

### 2.2 Data sources and case definition

Incident prostate cancer cases were extracted from the Unified Nationwide Electronic Health System using ICD-10 code C61 as the primary site. Duplicate records, non-resident cases, and records with unknown sex were excluded. Stage at diagnosis followed registry TNM conventions; for analysis we collapsed stage into I–II, III, IV, and unknown/unstaged. For morphological verification (MV) we used the registry variable indicating microscopic confirmation (histology/cytology) and calculated the proportion MV among all incident cases each year.

### 2.3 Population denominators

Annual male mid-year population estimates by 5-year age group and by region, including administrative details, were sourced from the Bureau of National Statistics of the Agency for Strategic Planning and Reforms of the Republic of Kazakhstan (10). These denominators were used to compute crude, age-specific, and age-standardized rates.

### 2.4 Statistical analysis

All incidence rates were calculated per 100,000 men and directly standardized to the WHO World Standard Population (2000–2025) using 5-year age groups (11). To eliminate the influence of the age structure of the male population in Kazakhstan on the incidence rate, age standardization was performed using the WHO World Standard Population. This approach adjusted for the comparatively younger demographic profile of the country, thereby ensuring that observed differences were not driven by population age composition. As a result, the age-standardized rate was higher than the crude incidence rate, which reflects the larger proportion of older age groups in the world standard population. This adjustment increases the calculated incidence value and allows for valid and comparable interpretation of the results in relation to global data.

For Joinpoint modelling, we worked on the log-rate scale and selected a log-linear model, assuming that the natural logarithm of the age-standardized rate changes linearly over time; the standard error (SE) of natural logarithm of the age-standardized incidence rate was calculated using the usual Poisson approximation based on case counts. For morphological verification (MV) %, standard errors were computed from binomial variance using the Wilson score formulation; these SEs were exported to Joinpoint when modelling MV as a proportion.

Temporal trends in overall and stage-specific incidence rates were estimated using the Joinpoint Regression Program (version 5.4.0, National Cancer Institute, Bethesda, MD, USA) (12). The primary model was chosen using the data-driven Weighted BIC among candidates with 0–3 joinpoints. To assess robustness, we also applied the Monte Carlo permutation test implemented in the Joinpoint Regression Program with an overall significance level α=0.05 and a maximum of 4,499 permutations (13). P-values for additional joinpoints were obtained from the empirical permutation distribution. To control the family-wise type-I error across multiple joinpoint tests, the software's built-in Bonferroni correction was used. The permutation-based selection yielded the same (or highly similar) number and location of joinpoints and comparable Annual Percent Change (APC)/Average Annual Percent Change (AAPC) estimates; therefore, the study conclusions were unchanged. 95% confidence intervals for APC/AAPC were computed via the Empirical Quantile method with 5,001 resamples. For MV (%), the same framework was applied to proportions with their SEs.

### 2.5 Ethics approval

This study utilized publicly available administrative data, and thus did not require direct interaction with individuals. Ethical approval was granted by the Local Ethics Commission of the Central Asian Institute for Medical Research.

## 3 Results

### 3.1 Incidence of prostate cancer in Kazakhstan (2005–2024)

During the period 2005–2024, 21,756 cases of prostate cancer were identified, representing an average annual number of 1209. The crude and standardized incidence rates were respectively of 12.6/100,000 men and 17.5/100,000 men (**Table 1**). The mean age at diagnostic was of 69.8 ± 0.2 years and the age specific incidence rate (ASIR) increased regularly with age.

**Table 1:** Prostate cancer in Kazakhstan 2005–2024.

Age group	Number (%)	ASIR*/100,000 men	95% CI**
0-4	2 (0.0)	0.01 ± 0.01	[0.00; 0.02]
5-9	0 (0.0)	0.00 ± 0.00	[0.00;0.00]
10-14	0 (0.0)	0.00 ± 0.00	[0.00;0.00]
15-19	0 (0.0)	0.00 ± 0.00	[0.00;0.00]
20-24	4 (0.0)	0.03 ± 0.01	[0.00;0.05]
25-29	3 (0.0)	0.02 ± 0.01	[0.00;0.05]
30-34	7 (0.0)	0.05 ± 0.02	[0.01;0.09]
35-39	11 (0.1)	0.09 ± 0.03	[0.04;0.14]
40-44	34 (0.2)	0.31 ± 0.08	[0.16;0.47]
45-49	152 (0.7)	1.47 ± 0.14	[1.20;1.75]
50-54	654 (3.0)	7.09 ± 0.83	[5.47;8.71]
55-59	1762 (8.1)	23.01 ± 1.81	[19.47;26.56]
60-64	3386 (15.6)	59.36 ± 4.48	[50.57;68.15]
65-69	5065 (23.3)	128.57 ± 11.09	[106.84;150.31]
70-74	4609 (21.2)	175.47 ± 9.85	[156.16;194.79]
75-79	3590 (16.5)	228.64 ± 15.13	[198.98;258.31]
80-84	1842 (8.5)	212.59 ± 12.91	[187.28;237.89]
85+	635 (2.9)	152.22 ± 7.56	[137.40;167.05]
Total	21756 (100.0)	–	
CIR		12.64 ± 0.85	[10.98;14.30]
ASR		17.46 ± 1.00	[15.50;19.43]

*ASIR, Age specific incidence rate, ***CI, Confidence interval.

### 3.2 Trends in incidence of prostate cancer in Kazakhstan (2005–2024)

**Figure 1:**
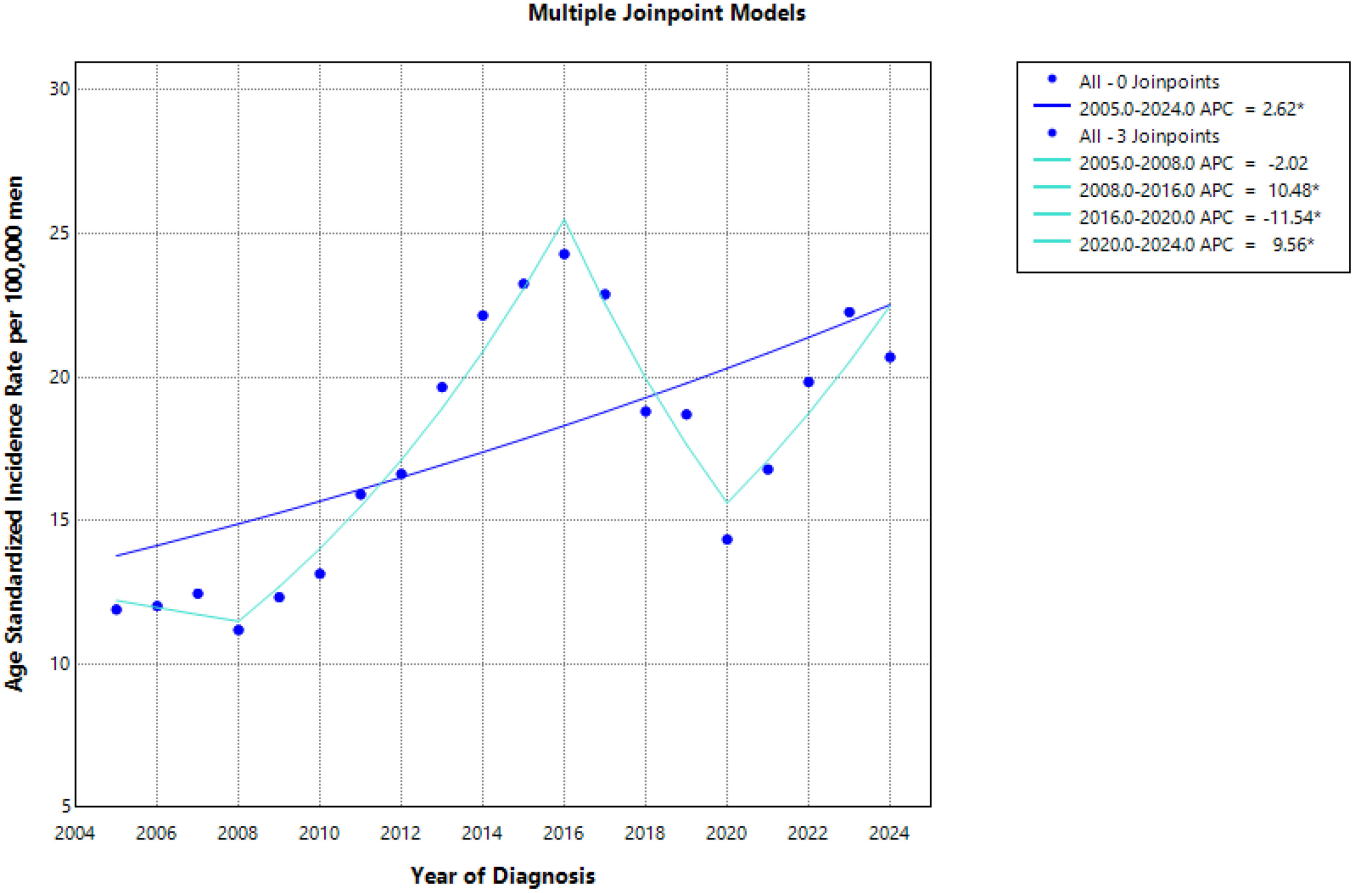
Trends in the age standardized incidence rate of prostate cancer for the period 2005-2024.

The ASR increased significantly from 11.9 in 2005 to 20.7 in 2024 with an APC of +2.6% (*p* = 0.002) (**Table 2**; **Figure 1**). Trends analysis has also described four periods: 2005–2008 APC of −2.0% (*p* = 0.731; not significant); 2008–2016 APC of +10.5% (*p* = 0.010); 2016–2020 APC of −11.5% (*p* = 0.005); and 2020–2024 APC of +9.6% (*p* = 0.002) (**Table 2**; **Figure 1**).

**Table 2:** Trends in the age standardized incidence rate of prostate cancer for the period 2005-2024.

Period	ASR*/100,000	APC (%)**	95% CI***	p****
2005-2024	11.9 – 20.7	+2.6	[1.0; 4.7]	0.002
2005-2008	11.9 – 11.2	−2.0	[−13.9; 8.2]	0.731
2008-2016	11.2 – 24.3	+10.5	[8.3; 19.1]	0.010
2016-2020	24.3 – 14.3	−11.5	[−18.5; −6.3]	0.005
2020-2024	14.3 – 20.7	+9.6	[3.7; 24.3]	0.002

*ASR, Age standardized incidence rate; **APC, Annual percentage change;

***CI, Confidence interval, ****p = level of significance.

### 3.3 Age-specific pattern

**Figure 2:**
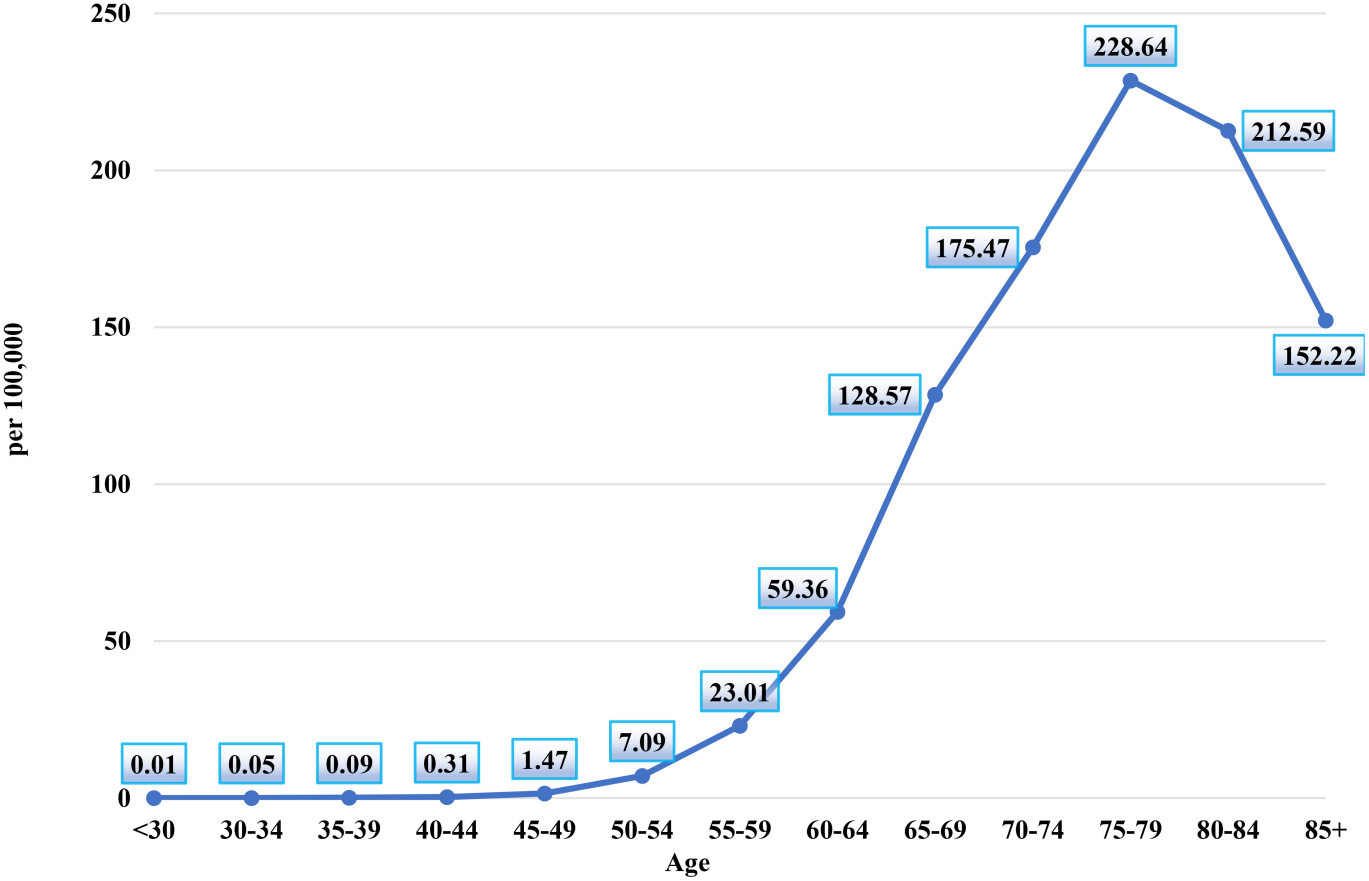
Age-specific prostate cancer incidence rates per 100,000 men, Kazakhstan, 2005–2024.

**Figure 2** illustrates the age-specific incidence rates of prostate cancer per 100,000 men in Kazakhstan between 2005 and 2024. The chart reveals a clear upward trend in prostate cancer incidence with advancing age, underscoring the disease's increasing prevalence among older male populations.

The chart shows that the incidence of prostate cancer is exceptionally low in younger age groups, with the rate in men under 30 years at 0.01 per 100,000. The incidence remains minimal in subsequent age groups, with the 30–34 years group at 0.05 per 100,000 and the 35–39 years group at 0.09 per 100,000, indicating that prostate cancer is rare in these early adult years.

A notable increase in incidence is observed beginning in the 40–44 years group, where the rate rises to 0.31 per 100,000, continuing to grow gradually through the 45–49 years (1.47 per 100,000) and 50–54 years (7.09 per 100,000) age groups.

The rate of increase accelerates in the older age brackets. The incidence reaches 23.01 per 100,000 men in the 55–59 years group, and further climbs to 59.36 per 100,000 in the 60–64 years group. The sharpest increase occurs between the 65–69 years and 70–74 years groups, with the incidence rising from 128.57 per 100,000 to 175.47 per 100,000.

The peak incidence rate is observed in the 75–79 years age group, where the rate reaches 228.64 per 100,000, marking the highest point on the chart. This indicates that prostate cancer incidence is most concentrated in men aged 75–79. Following this peak, the rate slightly decreases in the 80–84 years group (212.59 per 100,000) and again in the 85+ years group (152.22 per 100,000), suggesting a slight decline in incidence among the very elderly, though the rates remain high.

**Figure 3:**
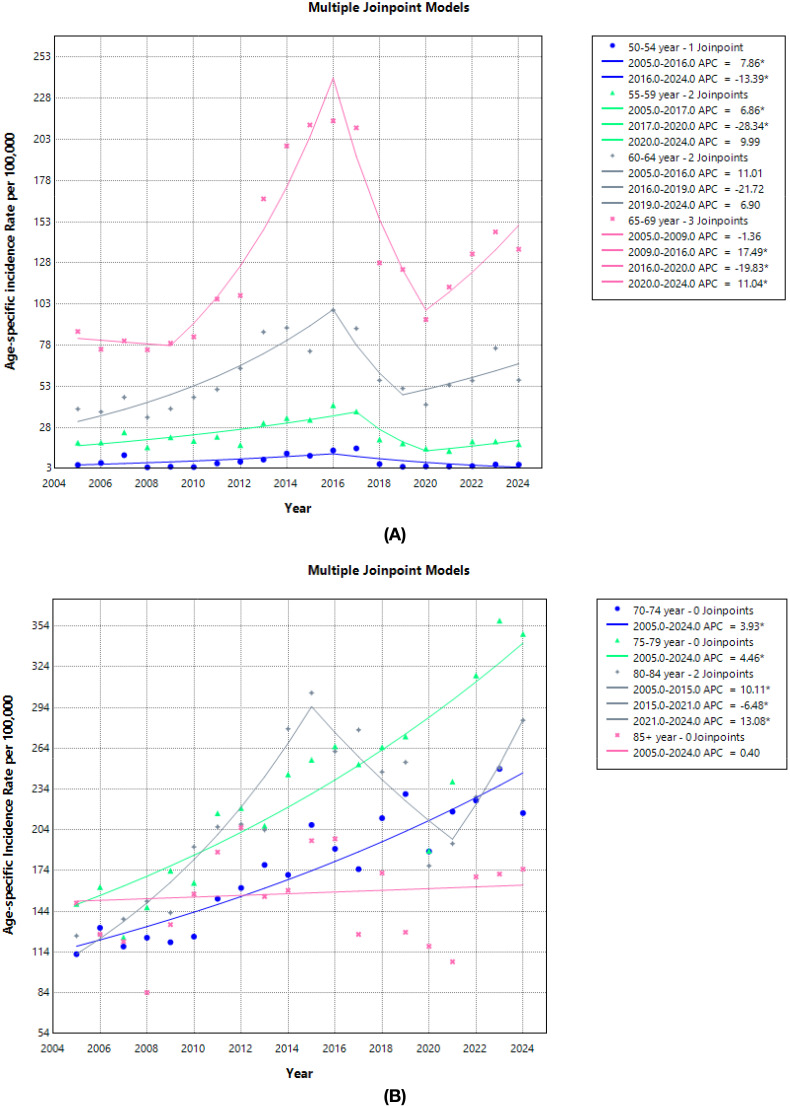
Trends in the age group specific incidence rate of prostate cancer cases for the period 2005-2024. **(A)** Trends in the age group (50-54, 55-59, 60-64, and 65-69) specific incidence rate of prostate cancer cases for the period 2005-2024. **(B)** Trends in the age group (70-74, 75-79, 80-44, and 85< year) specific incidence rate of prostate cancer cases for the period 2005-2024.

Across 2005–2024, age-specific incidence remained negligible in men <50 years (APC −2.0%/year; p=0.214). In 50–64 years the pattern was cyclical: a significant rise up to the mid-2010s (50–54: +7.9%/year; 55–59: +6.9%/year; 60–64: +11.0%/year), followed by a sharp downturn until 2019-2020 (−13.4%, −28.3%, and −21.7%/year, respectively) and a partial rebound thereafter. For 65–69 years, rates surged in 2009–2016 (+17.5%/year; p=0.004), dropped in 2016–2020 (−19.8%/year; p=0.001), and rose again in 2020–2024 (+11.0%/year; p=0.002) (**Figure 3A**; **Table 3**).

**Table 3:** Time trends of the age-specific incidence rates (2005 – 2024).

Age (years)	ASIR* (per 100,000)		APC (%)**	95% CI***	p****
	2005	2024			
< 50	0.2 ± 0.1	0.1 ± 0.0	2005-24: −2.0%	[−5.2; 1.3]	0.214
50-54	5.2 ± 1.2	5.4 ± 1.0	2005-16: +7.9% 2016-24: −13.4%	[2.1; 60.7] [−54.7; −4.6]	0.011 0.004
55-59	18.8 ± 2.7	17.9 ± 2.0	2005-17: +6.9% 2017-20: −28.3% 2020-24: +10.0%	[4.3; 10.9] [−36.5; −13.3] [−4.2; 45.6]	0.018 0.030 0.116
60-64	39.2 ± 5.1	56.8 ± 3.7	2005-16: +11.0% 2016-19: −21.7% 2019-24: +6.9%	[−4.1; 19.6] [−31.1; 29.6] [−4.9; 40.0]	0.064 0.098 0.162
65-69	86.3 ± 6.4	136.3 ± 6.8	2005-09: −1.4% 2009-16: +17.5% 2016-20: −19.8% 2020-24: +11.0%	[−19.3; 8.6] [13.7; 30.6] [−28.0; −12.5] [3.2; 27.3]	0.745 0.004 0.001 0.002
70-74	112.4 ± 10.7	216.4 ± 10.7	2005-24: +3.9%	[3.1; 5.0]	<0.001
75-79	149.5 ± 14.1	348.2 ± 20.4	2005-24: +4.5%	[3.2; 6.0]	<0.001
80-84	126.0 ± 21.9	284.6 ± 24.0	2005-15: +10.1% 2015-21: −6.5% 2021-24: +13.1%	[7.9; 13.2] [−13.2; −3.4] [4.7; 26.2]	0.001 0.001 0.001
85+	150.1 ± 37.5	175.1 ± 21.6	2005-24: +0.4%	[−1.4; 2.9]	0.489

*ASIR, age-specific incidence rate; **APC, Annual percentage change;

***CI,: Confidence interval, ****p = level of significance.

In older ages the burden increased more steadily. In 70–74 and 75–79 years the whole period showed significant growth (APC + 3.9% and +4.5%/year, respectively; both p<0.001). Among 80–84 years, a long increase in 2005–2015 (+10.1%/year; p=0.001) was followed by a mid-period dip (−6.5%/year; p=0.001) and renewed growth in 2021–2024 (+13.1%/year; p=0.001). The 85+ group was essentially stable (APC + 0.4%/year; p=0.489) (**Figure 3B**; **Table 3**).

### 3.4 Regional incidence rates and trends

**Figure 4:**
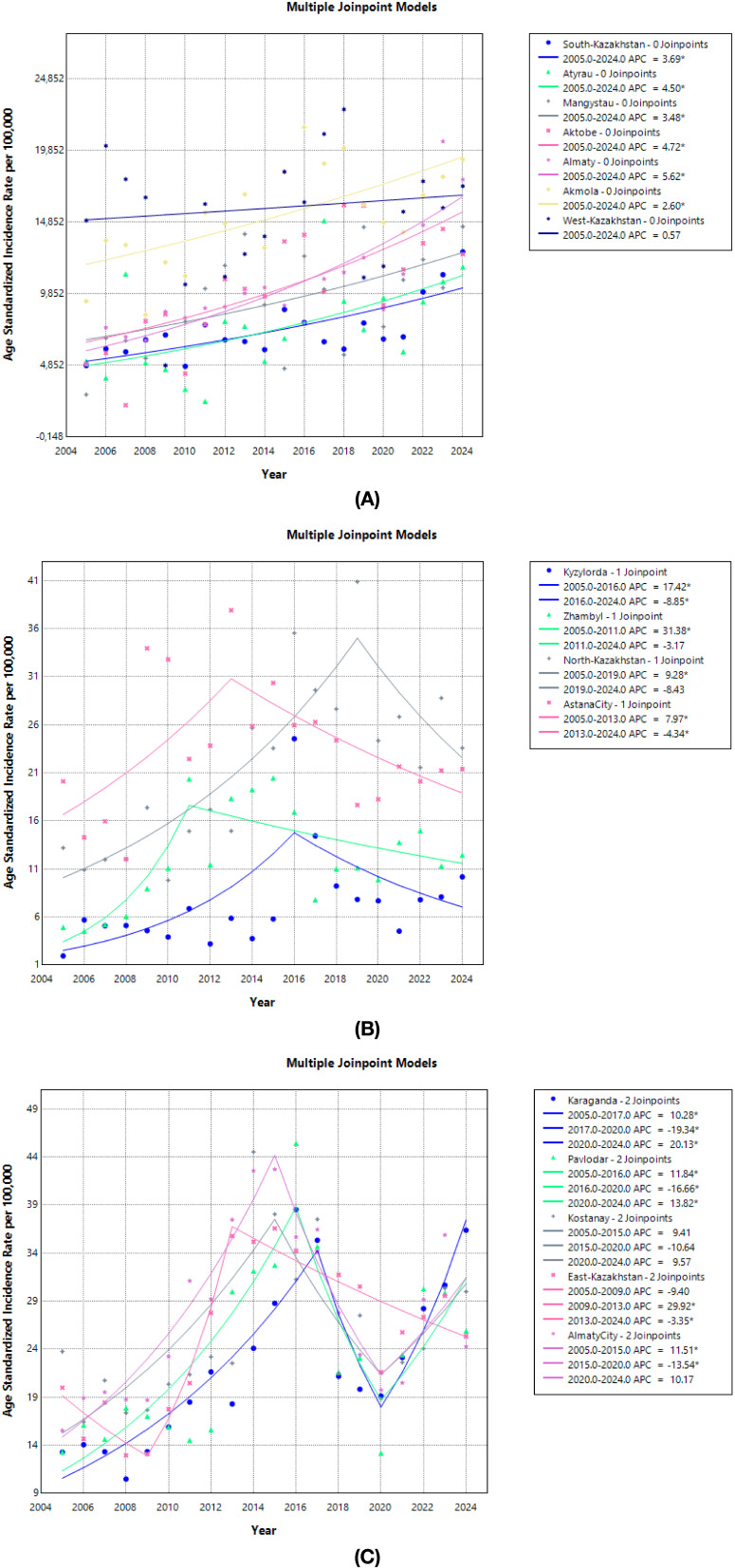
Age standardized incidence rate trends of prostate cancer by region, 2005-2024. **(A)** Age standardized incidence rate trends of prostate cancer by region (South-Kazakhstan, Atyrau, Mangystau, Aktobe, Almaty, Akmola, and West-Kazakhstan), 2005-2024. **(B)** Age standardized incidence rate trends of prostate cancer by region (Kyzylorda, Zhambyl, North-Kazakhstan, Astana City), 2005-2024. **(C)** Age standardized incidence rate trends of prostate cancer by region (Karaganda, Pavlodar, Kostanay, East-Kazakhstan, Almaty City), 2005-2024.

Monotonic, statistically significant increases were observed in South-Kazakhstan, Atyrau, Mangystau, Aktobe, Almaty (region), and Akmola (single-segment APCs ≈2.6 – 5.6%/year; p<0.05). West-Kazakhstan showed no significant long-term change (**Figure 4A**; **Table 4**).

**Table 4:** Age standardized incidence rate trends of prostate cancer by region, 2005-2024.

№	Region	Number of cases	ASR*/per 100,000 men	APC (%)**	95% CI***	p****
1	South-Kazakhstan	1005	7.3±0.5	2005-24: +3.7%	[2.0; 5.9]	<0.001
2	Atyrau	232	7.5±0.7	2005-24: +4.5%	[2.1; 7.8]	0.001
3	Mangystau	250	9.1±0.8	2005-24: +3.5%	[0.6; 7.9]	0.020
4	Aktobe	524	10.1±0.9	2005-24: +4.7%	[2.5; 7.8]	<0.001
5	Almaty	1479	10.3±0.9	2005-24: +5.6%	[4.2; 7.4]	<0.001
6	Akmola	1003	15.3±0.8	2005-24: +2.6%	[1.0; 4.7]	0.002
7	West-Kazakhstan	730	15.3±1.0	2005-24: +0.6%	[−1.5; 3.0]	0.535
8	Kyzylorda	321	7.3±1.1	2005-16: +17.4% 2016-24: −8.9%	[10.1; 73.4] [−32.8; −0.6]	0.001 0.036
9	Zhambyl	819	12.0±1.2	2005-11: +31.4% 2011-24: −3.2%	[15.0; 91.6] [−7.9; 0.2]	<0.001 0.063
10	North-Kazakhstan	1289	21.5±2.0	2005-19: +9.3% 2019-24: −8.4%	[6.7; 20.2] [−37.8; 1.6]	0.001 0.091
11	Astana city	958	23.3±1.5	2005-13: +8.0% 2013-24: −4.3%	[1.6; 45.5] [−17.3; −1.0]	0.012 0.012
12	Karaganda	2610	22.2±1.9	2005-17: +10.3% 2017-20: −19.3% 2020-24: +20.1%	[8.2; 14.0] [−25.9; −6.8] [11.7; 39.9]	0.002 0.002 0.001
13	Pavlodar	1561	23.4±2.1	2005-16: +11.8% 2016-20: −16.7% 2020-24: +13.8%	[8.1; 17.8] [−28.9; −5.0] [0.7; 39.6]	0.019 0.040 0.044
14	Kostanay	2166	25.5±1.8	2005-15: +9.4% 2015-20: −10.6% 2020-24: +9.6%	[−18.5; 31.5] [−28.0; 39.8] [−5.9; 45.9]	0.175 0.258 0.299
15	East-Kazakhstan	3498	25.6±1.8	2005-09: −9.4% 2009-13: +29.9% 2013-24: −3.3%	[−28.7; 0.9] [18.6; 47.4] [−5.3; −1.8]	0.072 <0.001 <0.001
16	Almaty city	3311	27.5±2.0	2005-15: +11.5% 2015-20: −13.5% 2020-24: +10.2%	[7.5; 17.5] [−27.1; −5.3] [−2.3; 38.1]	0.028 0.042 0.088

*ASR, Age standardized incidence rate; **APC, Annual percentage change; ***CI, Confidence interval, ****p = level of significance.

Regions with a rise followed by a significant decline included Kyzylorda (2005–2016 +17.4%/year; 2016–2024 −8.9%/year) and Astana city (2005–2013 +8.0%/year; 2013–2024 −4.3%/year). In Zhambyl and North-Kazakhstan the significant early increase (2005–2010 +31.4%/year and 2005–2019 +9.3%/year) was followed by a non-significant decrease (2011–2024 −3.2%/year and 2019–2024 −8.4%/year) (**Figure 4B**; **Table 4**).

"Boom–dip–rebound" profiles were evident in Karaganda (2005–2017 +10.3%/year; 2017–2020 −19.3%/year; 2020–2024 +20.1%/year), Pavlodar (2005–2016 +11.8%/year; 2016–2020 −16.7%/year; 2020–2024 +13.8%/year) and Almaty city (2005–2015 +11.5%/year; 2015–2020 −13.5%/year; 2020–2024 +10.2%/year). Kostanay showed non-significant changes across all segments. East Kazakhstan showed decline in 2005–2009, followed by increase in 2009–2013 (APC + 29.9%/year; p<0.001) and decrease in 2013–2024 (APC −3.3%/year; p<0.001) (**Figure 4C**; **Table 4**).

### 3.5 Trend of prostate cancer cases according to the extension stage

**Figure 5:**
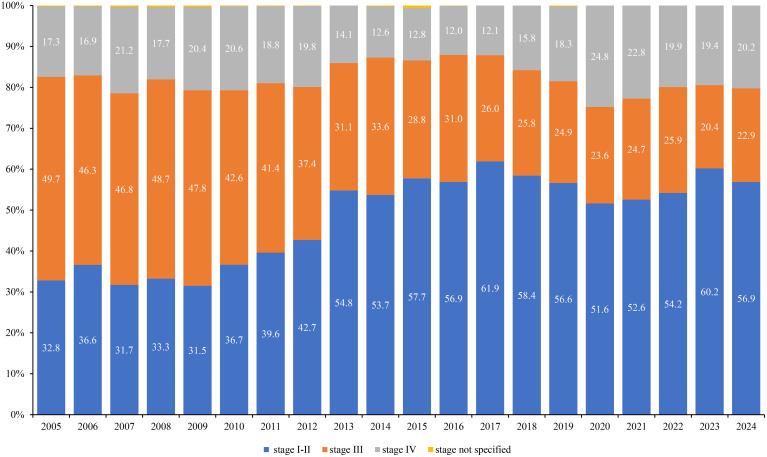
Dynamics of indicators of early diagnosis (stage I–II) and neglect (stage III and IV) of prostate cancer in Kazakhstan in 2005-2024.

From 2005 to 2024, the stage distribution shifted toward earlier diagnosis. The proportion at stage I–II rose from 32.8% to 56.9% (+24.1 percentage points), while stage III declined from 49.7% to 22.9% (−26.8 points); stage IV changed little overall (17.3% → 20.2%, +2.9 points). The share of stage I–II first surpassed stage III in 2012 and remained higher thereafter. Cases with unspecified stage fell from 0.17% to 0%. Across the entire period, the mean proportions were 53.0% for stage I–II (95% CI 49.0-56.9), 29.3% for stage III (95% CI 25.8-32.9), and 17.6% for stage IV (95% CI 15.5-19.7). Linear trends indicate strong trends for stage I–II (R²=0.72) and stage III (R²=0.92), with no meaningful linear trend for stage IV (R²=0.02) (**Figure 5**).

It is important to note that the concurrent rise in early-stage cases (stage I–II) alongside the relative stability of late-stage cases (stage IV) requires further considerations. Several factors could be influencing this trend, including potential lead-time bias, overdiagnosis, and diagnostic delays. Lead-time bias could cause an apparent increase in early-stage diagnoses due to the earlier detection of cases that would have previously presented at a later stage. Overdiagnosis might contribute to the rise in stage I–II diagnoses, particularly as more prostate cancers are detected through widespread screening that may not have otherwise caused symptoms or death. Additionally, diagnostic delays for more advanced cases might partially explain why the proportion of stage IV diagnoses has remained relatively constant, despite overall improvements in early detection.

### 3.6 Trends in prostate cancer incidence by stage and region

Nationally, the mean stage-specific incidence rates (per 100,000) were 6.3 ± 0.7 for stage I–II, 4.0 ± 0.2 for stage III, and 2.4 ± 0.1 for stage IV. Regionally, early-stage incidence was highest in East-Kazakhstan, North-Kazakhstan, Kostanay, and Pavlodar, and lowest in South-Kazakhstan, Mangystau, Atyrau and Kyzylorda; stage III was highest in Almaty city, Kostanay, Karaganda, and East-Kazakhstan and lowest in Atyrau, Kyzylorda, and Mangystau; stage IV was highest in Karaganda and Akmola and lowest in Kyzylorda, South-Kazakhstan and Atyrau (**Table 5**).

**Table 5:** Trends of prostate cancer incidence by stage and region, 2005-2024.

№	Regions	Stage I-II				Stage III				Stage IV			
		ASR*/per 100,000	APC**, %	95% CI ***	p****	ASR*/per 100,000	APC**, %	95% CI ***	p****	ASR*/per 100,000	APC**, %	95% CI ***	p****
1	Akmola	6.1±0.4	2005-24=+3.3%	[1.3; 5.7]	0.002	3.5±0.4	2005-18=+3.7% 2018-24=−12.5%	[−2.8; 116.7] [−62.7; 1.4]	0.100 0.066	4.8±0.7	2005-24=+9.8%	[7.2; 14.5]	<0.001
2	Aktobe	3.4±0.4	2005-24=+5.2%	[2.1; 10.0]	0.001	1.6±0.2	2005-24=+5.3%	[0.9; 11.3]	0.022	1.7±0.2	2005-24=+5.8%	[1.6; 12.3]	0.011
3	Almaty	3.4±0.6	2005-24=+12.2%	[10.4; 15.4]	<0.001	2.1±0.2	2005-18=−4.5% 2018-24=+9.8%	[−23.4; −0.2] [−1.0; 50.3]	0.048 0.079	2.0±0.2	2005-24=+6.5%	[4.6; 9.3]	<0.001
4	Atyrau	2.0±0.3	2005-24=+4.8%	[0.3; 11.8]	0.040	0.8±0.2	2005-24=+6.0%	[1.2; 14.2]	0.026	1.0±0.2	2005-24=+2.0%	[−1.8; 7.2]	0.262
5	East-Kazakhstan	15.8±2.0	2005-14=+29.7% 2014-24=−5.5%	[21.4; 50.8] [−11.5; −1.2]	<0.001 0.016	7.1±1.0	2005-11=−15.1% 2011-24=+10.1%	[−41.3; −2.6] [5.7; 25.5]	0.017 0.003	3.3±0.2	2005-24=+1.8%	[−0.7; 4.6]	0.152
6	Zhambyl	3.2±0.4	2005-24=+10.0%	[7.4; 14.8]	<0.001	1.6±0.3	2005-24=−1.6%	[−5.2; 2.1]	0.359	1.3±0.2	2005-07=+4.5% 2007-24=+17.9%	[4.5; 4.5] [14.1; 43.0]	<0.001 <0.001
7	West-Kazakhstan	8.9±0.8	2005-24=−0.2%	[−4.6; 4.4]	0.967	4.0±0.7	2005-18=+10.2% 2018-24=−22.6%	[4.4; 30.7] [−67.5; −7.1]	0.003 0.003	1.8±0.3	2005-18=−3.5% 2018-24=+20.2%	[−36.3; 50.9] [1.8; 88.9]	0.244 0.036
8	Karaganda	7.0±1.4	2005-24=+13.0%	[9.4; 20.7]	<0.001	8.2±0.9	2005-16=+8.6% 2016-24=−10.3%	[4.3; 18.1] [−24.6; −4.6]	<0.001 <0.001	5.2±0.5	2005-24=+3.5%	[−0.3; 8.2]	0.074
9	Kostanay	14.3±2.3	2005-24=+8.7%	[4.6; 15.8]	<0.001	9.0±1.1	2005-12=+8.9% 2012-24=−6.9%	[0.5; 59.0] [−23.4; −3.2]	0.033 0.005	2.2±0.2	2005-17=−6.3% 2017-24=+5.9%	[−20.2; −3.3] [−2.0; 41.2]	0.017 0.182
10	Kyzylorda	2.7±0.8	2005-16=+30.6% 2016-24=−18.7%	[19.9; 97.6] [−43.6; −9.4]	<0.001 0.004	0.8±0.1	2005-24=+4.0%	[0.8; 8.3]	0.016	0.8±0.2	2005-24=+9.2%	[6.2; 13.7]	<0.001
11	Mangystau	1.7±0.2	2005-24=+6.7%	[3.3; 12.7]	0.001	1.1±0.2	2005-24=+0.1%	[−4.4; 5.7]	0.818	1.4±0.2	2005-24=+10.4%	[6.0; 18.4]	<0.001
12	Pavlodar	14.2±1.9	2005-24=+5.8%	[2.2; 10.8]	0.004	5.1±0.7	2005-24=+7.0%	[4.5; 10.7]	<0.001	2.8±0.4	2005-12=+11.1% 2012-24=−10.7%	[2.0; 39.4] [−21.0; −6.6]	0.023 <0.001
13	North-Kazakhstan	15.4±2.5	2005-24=+10.5%	[6.4; 17.8]	<0.001	4.6±0.5	2005-24=−3.2%	[−6.1; −0.8]	0.012	3.5±0.3	2005-24=+7.0%	[4.7; 10.3]	<0.001
14	South-Kazakhstan	0.9±0.1	2005-24=+0.4%	[−3.9; 5.6]	0.735	1.9±0.2	2005-21=−1.5% 2021-24=+24.7%	[−20.4; 27.5] [0.1; 74.3]	0.303 0.050	0.9±0.2	2005-24=+19.1%	[14.3; 27.8]	<0.001
15	Almaty city	9.0±1.1	2005-24=+5.8%	[2.4; 11.5]	0.004	9.0±1.4	2005-15=+9.9% 2015-24=−17.5%	[4.8; 18.7] [−25.2; −13.1]	<0.001 <0.001	3.6±0.3	2005-24=+4.7%	[3.1; 7.0]	<0.001
16	Astana city	5.9±0.4	2005-16=+6.1% 2016-19=−28.2% 2019-24=+17.7%	[3.2; 12.4] [−36.8; −11.3] [8.1; 48.9]	0.003 0.004 0.004	2.0±0.2	2005-24=−4.0%	[−7.2; −0.3]	0.036	2.8±0.2	2005-24=+4.6%	[1.5; 9.8]	0.009
17	Kazakhstan	6.3±0.7	2005-09 = 0.0% 2009-16=+22.8% 2016-20=−14.9% 2020-24=+15.3%	[−22.9; 13.2] [18.4; 36.3] [−23.1; −7.4] [7.4; 31.7]	0.970 0.004 0.003 <0.001	4.0±0.2	2005-16=+3.1% 2016-20=−13.5% 2020-24=+9.8%	[1.4; 5.8] [−22.9; −6.3] [0.1; 31.3]	0.020 0.024 0.048	2.4±0.1	2005-24=+4.7%	[4.0; 5.6]	<0.001

*ASR, Age standardized incidence rate; **APC, Annual percentage change; ***CI, Confidence interval, ****p = level of significance.

At the national level, stage I–II incidence increased in 2009–2016, declined in 2016–2020, and increased again in 2020–2024; stage III rose until 2016, declined in 2016–2020, and rose slightly in 2020–2024; stage IV increased throughout 2005–2024. Regionally, stage I–II increased monotonically in Akmola, Aktobe, Almaty (region), Atyrau, Zhambyl, Karaganda, Kostanay, Mangystau, Pavlodar, North-Kazakhstan, and Almaty city, showed no change in West-Kazakhstan and South-Kazakhstan, and followed segmented patterns in East-Kazakhstan (increase to 2014, decrease to 2024) and Astana city (increase to 2016, decrease to 2019, increase to 2024). For stage III, long-term decreases were observed in Zhambyl, North-Kazakhstan, and Astana city; increases in Aktobe, Atyrau, Pavlodar, and Kyzylorda; and no long-term change in Mangystau. Rise-then-fall trajectories occurred in Akmola, West-Kazakhstan, Karaganda, Kostanay, and Almaty city, whereas fall-then-rise patterns were seen in Almaty (region), East-Kazakhstan, and South-Kazakhstan. For stage IV, statistically significant long-term increases were observed in Akmola, Aktobe, Almaty (region), Kyzylorda, Mangystau, North-Kazakhstan, South-Kazakhstan, Almaty city, and Astana city. No significant long-term change was detected in Atyrau, East-Kazakhstan, Karaganda. Zhambyl exhibited two significant increasing segments. West-Kazakhstan had a non-significant decline to 2018 followed by a significant increase in 2018–2024. Pavlodar increased to 2012 and then declined significantly thereafter. Kostanay declined significantly to 2017 and then increased non-significantly in 2017–2024 (**Table 5**).

### 3.7 Regional patterns in morphological verification rates

**Figure 6:**
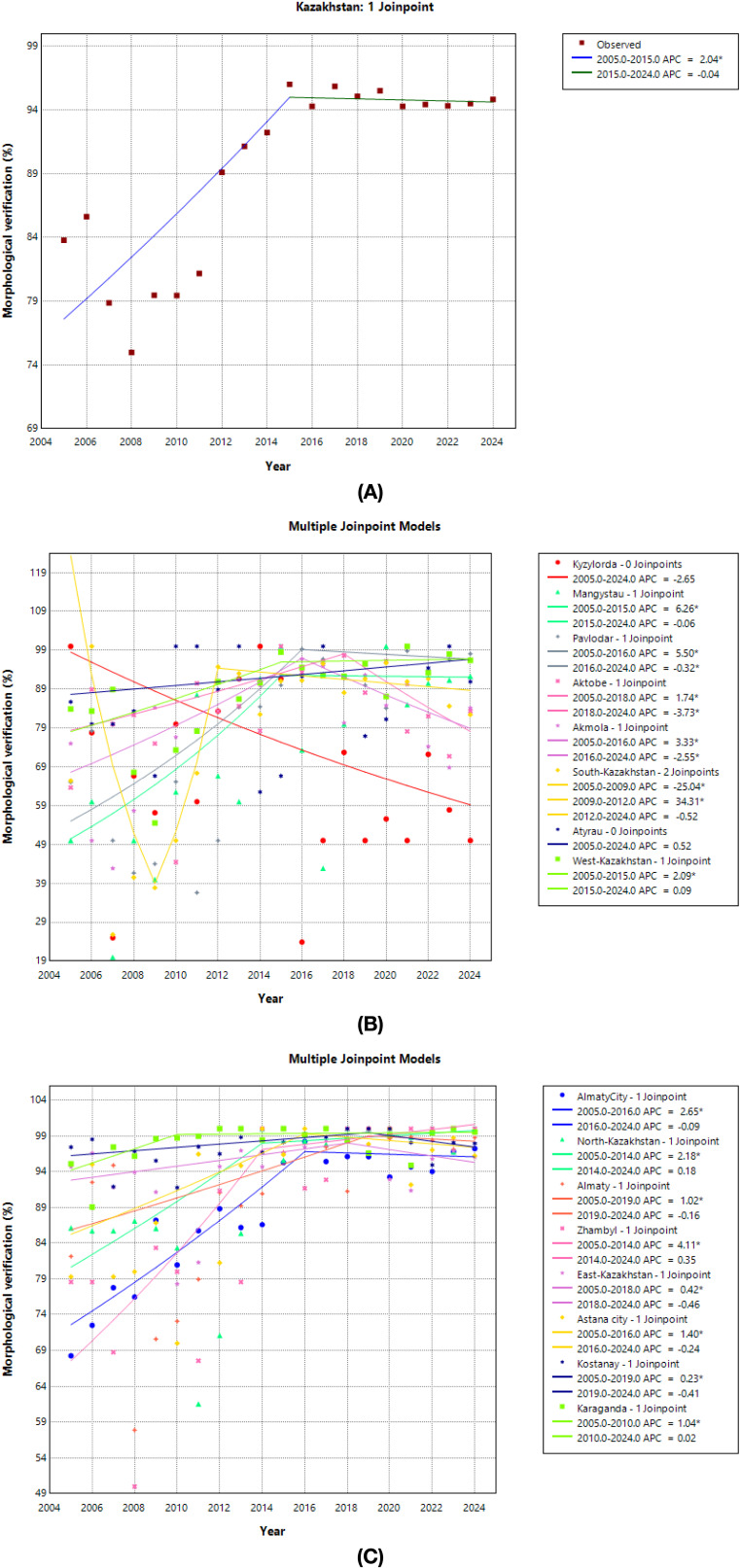
Trends of morphological verification rates by regions. **(A)** Trends of Morphological Verification Rates in Kazakhstan. **(B)** Trends of Morphological Verification Rates by Regions (Kyzylorda, Mangystau, Pavlodar, Aktobe, Akmola, South-Kazakhstan, Atyrau, and West-Kazakhstan). **(C)** Trends of Morphological Verification Rates by Regions (Almaty City, North-Kazakhstan, Almaty, Zhambyl, East-Kazakhstan, Astana City, Kostanay, and Karaganda).

Nationally, morphological verification was high (mean ≈92%) and improved during 2005–2015 (APC + 2.0%/year, *p* < 0.001), then stabilized with no further change in 2015–2024 (APC −0.04%/year) (**Figure 6A**; **Table 6**).

**Table 6:** Morphological verification trends of prostate cancer by region, 2005-2024.

№	Region	MV* (%)±m	APC**, %	95% CI***	p****
1	Kyzylorda	65.9±5.6	2005-24=−2.6%	[−5.7; 0.4]	0.092
2	Mangystau	81.3±4.5	2005-15=+6.3% 2015-24=−0.1%	[2.8; 106.5] [−6.5; 2.3]	0.006 0.940
3	Pavlodar	84.0±5.0	2005-16=+5.5% 2016-24=−0.3%	[5.5; 11.4] [−1.5; −0.1]	<0.001 0.016
4	Aktobe	84.5±3.7	2005-18=+1.7% 2018-24=−3.7%	[1.1; 5.5] [−10.3; −1.7]	<0.001 0.002
5	Akmola	85.8±2.2	2005-16=+3.3% 2016-24=−2.5%	[2.0; 11.4] [−8.5; −0.7]	<0.001 0.009
6	South-Kazakhstan	86.0±3.3	2005-09=−25.0% 2009-12=+34.3% 2012-24=−0.5%	[−48.6; −17.1] [14.8; 65.9] [−1.7; 0.5]	0.002 0.002 0.274
7	Atyrau	89.7±3.3	2005-24=+0.5%	[−0.3; 2.2]	0.123
8	West-Kazakhstan	91.2±2.0	2005-15=+2.1% 2015-24=+0.1%	[1.4; 14.3] [−2.7; 0.8]	0.005 0.999
**9**	**Kazakhstan**	**92.1±1.4**	**2005-15=+2.0%** **2015-24=−0.04%**	**[1.5; 3.2]** **[−0.5; 0.3]**	**<0.001** **0.717**
10	Almaty city	92.3±1.4	2005-16=+2.6% 2016-24=−0.1%	[2.3; 4.6] [−0.7; 0.3]	<0.001 0.575
11	North-Kazakhstan	92.6±3.2	2005-14=+2.2% 2014-24=+0.2%	[1.3; 8.3] [−0.7; 0.5]	<0.001 0.659
12	Almaty	93.4±2.1	2005-19=+1.0% 2019-24=−0.2%	[0.3; 15.1] [−2.2; 1.0]	0.040 0.847
13	Zhambyl	93.5±2.7	2005-14=+4.1% 2014-24=+0.4%	[2.6; 10.3] [−0.5; 1.0]	<0.001 0.270
14	East-Kazakhstan	93.8±1.6	2005-19=+0.4% 2019-24=−0.5%	[0.2; 3.5] [−4.4; 0.1]	0.013 0.087
15	Astana city	94.6±2.2	2005-16=+1.4% 2016-24=−0.2%	[0.8; 10.4] [−3.5; 0.4]	0.004 0.481
16	Kostanay	97.8±0.6	2005-19=+0.2% 2019-24=−0.4%	[0.1; 2.2] [−2.8; 0.0]	0.018 0.087
17	Karaganda	98.9±0.4	2005-10=+1.0% 2010-24=+0.02%	[0.1; 5.7] [−0.4; 0.2]	0.040 0.618

*MV, Morphological Verification rate; **APC, Annual percentage change;

***CI, Confidence interval, ****p = level of significance.

The bold row is morphological verification trend is the country of Kazakhstan.

Across regions, five reproducible patterns emerged:

1. *Ceiling and stable* (mean ≥97%): Karaganda (mean ≈99%) and Kostanay (≈98%) showed only minimal early increases, then flat trends thereafter (**Figure 6C**; **Table 6**).
2. *High with modest-to-substantial early improvement then plateau* (means ~92–95%): early gains were steeper in Almaty city and North-Kazakhstan (APC of +2.6% and +2.2%/year, *p* < 0.001), modest in East-Kazakhstan, Almaty region and Astana city (APC of +0.4%, +1.0% and 1.4%/year, *p* < 0.05), and substantial in Zhambyl (+4.1%/year to 2014, *p* < 0.001), followed by stable trends thereafter (**Figure 6C**; **Table 6**).
3. *Moderate catch-up then softening* (means ~81–91%): Pavlodar, Aktobe, Akmola rose significantly and later showed declines; Mangystau rose steeply to 2015 and then flattened; Atyrau showed no long-term change; West-Kazakhstan increased modestly to 2015 then plateaued (**Figure 6B**; **Table 6**).
4. *Transitional/oscillatory*: South-Kazakhstan declined sharply in 2005–2009, rebounded in 2009–2012, then remained stable (**Figure 6B**; **Table 6**).
5. *Persistently low outlier:* Kyzylorda maintained the lowest morphological verification (mean ≈ 66%) with a non-significant downward tendency (**Figure 6B**; **Table 6**).

By the late 2010s, most regions converged to very high morphological verification (≈90–99%), mirroring the national plateau.

## 4 Discussion

From 2005 to 2024, a total of 21,756 cases of prostate cancer were identified in Kazakhstan. The crude and standardized incidence rates were 12.6 and 17.5 per 100,000 men, respectively. The mean age at diagnosis was 69.8 years, and the age-specific incidence rate (ASIR) showed a consistent increase with age. The ASIR significantly rose from 11.9 in 2005 to 20.7 in 2024, with an annual percent change (APC) of +2.6% (p=0.002). Trends revealed distinct periods: a slight decline from 2005–2008, a sharp increase from 2008–2016, a notable decline from 2016–2020, and a rebound from 2020–2024. Age-specific incidence remained negligible in men under 50 years and showed cyclical patterns in those aged 50–64, with steady increases in older age groups. Geographically, regions like South-Kazakhstan, Atyrau, Mangystau, and Aktobe experienced steady increases, while West-Kazakhstan showed no significant long-term change. The incidence of prostate cancer staged earlier in the study period, with the proportion of stage I–II cases rising significantly from 32.8% to 56.9%, while stage III decreased from 49.7% to 22.9%. The national morphological verification rate remained high, averaging 92%, with regional variations reflecting substantial improvements in most areas.

### 4.1 Prostate cancer incidence: global context

Globally, prostate cancer incidence has shown substantial variation across regions, with developed countries, particularly those in North America, Northern Europe, and Oceania, experiencing the highest rates (14). Schafer et al. reported that the age-standardized incidence rate (ASIR) for prostate cancer in the world reached 29.4 per 100,000 in 2022 (14).

In the United States, prostate cancer age-adjusted incidence rates decreased from 165.8 per 100,000 in 2007 to 101 per 100,000 in 2014 (APC=-6.51, p*<*0.05) and then increased to 121.2 per 100,000 in 2021 (APC = 1.87, p*<* 0.05) (15). Similarly, Gomez et al. found that the incidence of localized prostate cancer increased by 3.7% annually from 2014 to 2017 (16).

In Kazakhstan, the prostate cancer incidence has mirrored global trends, with a notable increase over the past two decades. The data from Kazakhstan's prostate cancer registry show that the age-standardized incidence rate (ASIR) rose from 11.9 per 100,000 in 2005 to 20.7 per 100,000 in 2024, reflecting a 2.6% annual increase. Similar to global trends, this increase can likely be attributed to improvements in healthcare infrastructure, enhanced screening, and greater awareness of the disease. However, Kazakhstan's incidence rate remains lower than that of high-income countries, such as the United States, where the incidence reached 121.2 per 100,000 in 2021 (14).

### 4.2 Age-specific incidence patterns and trends

Age-specific incidence rates reveal that prostate cancer incidence increases significantly with age, a trend observed both in Kazakhstan and globally. Jacklin et al. noted that older men are more likely to be diagnosed with prostate cancer due to the nature of the disease's progression (17), and Kazakhstan's findings corroborate this, with the highest incidence observed in men aged 75–79 years, where the rate was 228.64 per 100,000. This trend aligns with findings from California, United States, where Van Blarigan et al. (18) reported that 92.1% of prostate cancer cases were among men aged 55 and older. Between 2004 and 2021, California recorded 387,636 prostate cancer cases, including 27,938 at the distant stage, and 58,754 prostate cancer-related deaths. In this cohort, 203,038 cases (52.4%) occurred in men aged 55 to 69, while 153,884 cases (39.7%) were diagnosed in men aged 70 and above (18).

### 4.3 Regional variations in prostate cancer incidence

Regional disparities in prostate cancer incidence are evident in both Kazakhstan and globally, and they are often driven by differences in healthcare access and socioeconomic factors. Seikkula et al. found that in Finland, urban area have higher incidence rate of prostate cancer than rural area (19).

A similar regional pattern was observed in Kazakhstan. Urban centers like Almaty and Karaganda reported higher prostate cancer incidence, likely due to better access to screening programs and healthcare services. In contrast, rural regions such as South Kazakhstan and Mangystau exhibited lower incidence rates, which could indicate underdiagnosis or delayed diagnoses of prostate cancer.

### 4.4 Covid-19 pandemic effect on prostate cancer incidence rate

Although Kazakhstan-specific data on PSA screening uptake and diagnostic throughput are not available, the observed decline in incidence between 2016 and 2020, followed by a rebound after 2020, is temporally compatible with contraction and subsequent partial recovery of oncological service activity during and after the COVID-19 pandemic. Similar diagnostic disruptions were widely reported worldwide. According to Soerjomataram et al., 65.6% of population-based cancer registries across 90 countries experienced interruptions in case reporting, data collection, and pathology verification due to pandemic-related restrictions. These disruptions likely resulted in under-registration of new cancer cases and delayed diagnosis globally (20).

More specifically for prostate cancer, Mangone et al. conducted a population-based analysis in Northern Italy and demonstrated a 31% reduction in newly diagnosed prostate cancer cases in 2020 (21). Taken together, these factors suggest that the temporary downturn in prostate cancer incidence in 2016–2020 and its rebound after 2020 may represent a compound effect of (a) reduced diagnostic and screening activity during the pandemic, (b) delayed registration and patient flow disruptions due to national quarantine measures, and (c) gradual restoration of service capacity in 2021–2022. This interpretation aligns with international evidence on COVID-19-induced diagnostic backlogs and supports the inclusion of pandemic-related contextual factors when interpreting prostate cancer incidence trends in Kazakhstan.

### 4.5 Strengths and limitations

#### 4.5.1 Strengths

This study utilizes a comprehensive nationwide dataset covering a 20-year period (2005–2024), facilitating a thorough evaluation of long-term trends and regional variations in prostate cancer incidence. Age-standardized rates were calculated according to international standards, and age-specific patterns were meticulously delineated. Using Joinpoint segmented regression gives clear estimates of period-specific APCs and AAPCs, which show when incidence "bump-dip-rebound" patterns happen. Stage-specific and morphological-verification analyses enhance the validity of registry signals and augment the study's policy relevance by correlating temporal trends with diagnostic and screening dynamics.

#### 4.5.2 Limitations

The analysis is based on registry data, which makes it ecological. Individual-level factors like PSA testing history, biopsy results, Gleason grade, comorbidities, and treatment are not available, which makes it hard to draw conclusions about the effects of screening and outcomes.

Changes in diagnostic practice, staging, or coding may explain some of the temporal trends, rather than real changes in disease occurrence.

Several joinpoint segments only cover a few years and small counts, so short-term APC estimates should be taken with a grain of salt. Even though morphological verification is high nationally, there is meaningful regional variability (notably lower %MV in some regions), which could lead to different ascertainment across areas. Finally, the lack of linked mortality and survival data makes it impossible to see if earlier-stage detection led to better outcomes; pandemic-era disruptions around 2020–2021 may also make it hard to interpret trends.

## 5 Conclusion

In sum, Kazakhstan exhibits growing incidence with period inflections, a national shift toward earlier stage at diagnosis, and concurrent increases in stage IV, set against high and converging morphological verification. These features align with global narratives on PSA dynamics and pandemic disruptions, while regional typologies identify where early-detection gains are durable and where late-stage pressure persists. Moving from description to attribution will require formal difference-in-differences/event-study designs and sensitivity analyses that exclude 2020–2021, alongside audits of screening uptake, diagnostic capacity, and time-to-biopsy/treatment to ensure that earlier detection translates into better outcomes.

## Data Availability

The datasets presented in this study can be found in online repositories. The names of the repository/repositories and accession number(s) can be found in the article/Supplementary Material.
